# L-lysine potentiates aminoglycosides against *Acinetobacter baumannii* via regulation of proton motive force and antibiotics uptake

**DOI:** 10.1080/22221751.2020.1740611

**Published:** 2020-03-20

**Authors:** Wanyan Deng, Tiwei Fu, Zhen Zhang, Xiao Jiang, Jianping Xie, Hang Sun, Peng Hu, Hong Ren, Peifu Zhou, Qi Liu, Quanxin Long

**Affiliations:** a Key Laboratory of Molecular Biology for Infectious Diseases (Ministry of Education), Institute for Viral Hepatitis, Department of Infectious Diseases, The Second Affiliated Hospital of Chongqing Medical University, Chongqing, PR People’s Republic of China; b Chongqing Key Laboratory for Oral Diseases and Biomedical Sciences, Chongqing Municipal Key Laboratory of Oral Biomedical Engineering of Higher Education, Stomatological Hospital of Chongqing Medical University, Chongqing, People’s Republic of China; c Department of Clinical Laboratory, Chongqing General Hospital, Chongqing, People’s Republic of China; d State Key Laboratory Breeding Base of Eco-Environment and Bio-Resource of the Three Gorges Area, Key Laboratory of Eco-environments in Three Gorges Reservoir Region, Ministry of Education, School of Life Sciences, Institute of Modern Biopharmaceuticals, Southwest University, Chongqing, People’s Republic of China; e School of Ethnic-Minority Medicine, Guizhou Minzu University, Guizhou, People’s Republic of China

**Keywords:** L-Lysine, aminoglycosides, *Acinetobacter baumannii*, proton motive force, reactive oxygen species

## Abstract

*Acinetobacter baumannii*, a Gram-negative opportunistic pathogen, is a leading cause of hospital- and community-acquired infections. *Acinetobacter baumannii* can rapidly acquire diverse resistance mechanisms and undergo genetic modifications that confer resistance and persistence to all currently used clinical antibiotics. In this study, we found exogenous L-lysine sensitizes *Acinetobacter baumannii*, other Gram-negative bacteria (*Escherichia coli* and *Klebsiella pneumoniae*) and a Gram-positive bacterium (*Mycobacterium smegmatis*) to aminoglycosides. Importantly, the combination of L-lysine with aminoglycosides killed clinically isolated multidrug-resistant *Acinetobacter baumannii* and persister cells. The exogenous L-lysine can increase proton motive force via transmembrane chemical gradient, resulting in aminoglycoside acumination that further accounts for reactive oxygen species production. The combination of L-lysine and antibiotics highlights a promising strategy against bacterial infection.

## Introduction

*Acinetobacter baumannii* (*A. baumannii*) is a notorious nosocomial infection pathogen and a major source of hospital-acquired pathogen in intensive care units (ICUs) [1–3]. It provokes a 26% and 43% mortality rate in hospital settings and ICUs, respectively [1]. In addition, most clinical *A. baumannii* isolates are naturally competent [4], and they can rapidly acquire genetic elements for drug resistance and undergo genetic modifications that make bacteria resistant to all currently used clinical antibiotics [5–7]. Therefore, it is particularly problematic and extremely difficult to treat *A. baumannii* infection. From the recently published Tigecycline Evaluation and Surveillance Trial (TEST) data, 44% of *A. baumannii* shows multidrug-resistant (MDR) characteristics, which is the highest rate among Gram-negative pathogens [8]. Because of this, multidrug-resistant *A. baumannii* especially carbapenem-resistant *A. baumannii* is classified at a threat level of “urgent” pathogen by the United States Centers for Disease Control and Prevention. It requires more and urgent attention for discovering new antibiotics or adjuvant for antibiotics against *A. baumannii* infections [9–12].

Aminoglycoside antibiotics (AGs) are widely used to treat Gram-negative infections including *Escherichia coli* (*E. coli*), *Enterobacteriaceae spp*, *Pseudomonas aeruginosa* (*P. aeruginosa*), *Klebsiella pneumoniae* (*K. pneumoniae*) and *Acinetobacter baumannii* (*A. baumannii*), and some Gram-positive infections [13]. During AG treatment, diverse mechanisms, including inactivation of drug target, toxin–antitoxin modules and drug efflux pumps-induced multidrug resistant bacteria (MDR) and persister formation, can cause chronic bacterial infection [14–17]. One of the typical examples of resistance is that any influence on the bacterial proton motive force (PMF), including Δψ (the electrical potential across the membrane) and ΔpH (the transmembrane difference in the H^+^ concentration) can significantly change the bactericidal activity of AGs [18]. Novel approaches that target this phenotypic resistance mechanism to restore the cell’s initial susceptibility to antibiotics may be an alternative approach to solve drug resistance [19–23]. Specific metabolites, such as glucose, fructose, mannitol, or pyruvate, promote NADH production through glycolysis, resulting in an increased PMF production through the electron transport chain [24–26]. Jean-Marc Ghigo et al reported that the effect of basic amino acid L-arginine on gentamicin against bacterial infection probably relies on the ΔpH-dependent transmembrane difference in the H^+^ ion concentration rather than membrane electrical potential [27], suggesting pH-mediated potentiation of AGs is effective against nosocomial pathogens.

An interesting study has demonstrated that L-lysine could enhance the antifungal effect of Amphotericin B (AmB) against fungal infections, such as *Candida albicans*, *Candida parapsilosis* and *Cryptococcus neoformans*, compared with AmB alone [28]. Schweizer and coworkers described a novel class of tobramycin-lysine conjugates containing an optimized amphiphilic tobramycin-C12 tether that sensitizes Gram-negative bacteria to legacy antibiotics [29]. These studies provide a promising strategy for the therapy of fungal and bacterial infection by using antibiotics together with L-lysine. Whether L-lysine could potentiate AGs against bacteria, especially drug-resistant bacteria, needs to be elucidated. In this study, we found that exogenous L-lysine stimulates the bactericidal ability of AGs against *A. baumannii* (persisters and clinical MDR strains). The mechanism underlying this process is that L-lysine promotes transmembrane pH difference (ΔpH), which, in turn, increases PMF and stimulates uptake of AGs. During antibiotic stress, endogenous reactive oxygen species (ROS) is also induced simultaneously to kill the bacteria. Importantly, L-lysine increases the efficiency of AGs against both a Gram-negative bacteria (*E. coli* and *K. pneumoniae*) and a Gram-positive bacterium *Mycobacterium smegmatis* (*M. smegmatis*). In summary, this work establishes a strategy for eradicating multidrug-resistant bacteria and bacterial persisters, highlighting the synergistic effects of L-lysine on antibiotics against bacterial infection.

## Materials and methods

### Strains and culturing conditions

*A. baumannii* ATCC19606 and clinically isolated multidrug-resistant *A. baumannii* 18030945 and 16010214, *E. coli* ATCC 25922, *K. pneumoniae* ATCC700603 and *M. smegmatis* MC^2^-155 were used in this study. *M. smegmatis* MC^2^-155 was grown in Middlebrook 7H9 broth medium supplemented with 0.05% Tween 80 and 0.5% glycerol. The experimental and stationary phase bacteria were grown in the following way: bacteria from frozen stock were grown at 37°C, 220 rpm in Luria–Bertani (LB) broth overnight. Cells were then diluted 1:1000 in 50 ml LB to an optical density (OD_600_) of 0.6 or grown for 16 h at 37°C, 250 rpm in 250 mL flasks, respectively. The cultures were washed with phosphate buffer solution (PBS), suspended in M9 minimal medium supplemented with 10 mM acetate, 1 mM MgSO_4_ and 100 mM CaCl_2_ (referred to M9 medium in the text) and treated with different antibiotics with or without L-lysine using the indicated concentrations.

### Antibiotics and chemicals

Kanamycin (Kan), gentamicin (Gen) and amikacin (Ami) were purchased from Sangon Biotech Co. (Shanghai, China), and their stock solutions were freshly prepared, filter-sterilized and used at the indicated concentrations. L-lysine was obtained from Sigma-Aldrich (USA). A stock solution of amino acids (2 mM) was prepared in ddH_2_O, stored at −20°C and used at the indicated concentrations. Carbonyl cyanide 3-chlorophenylhydrazone (CCCP), bis-(1,3-dibutylbarbituric acid) trimethine oxonol (DiBAC4(3)), 2′,7′-dichlorofluorescin diacetate (DCFH-DA) and dimethyl sulfoxide (DMSO) were obtained from Sigma-Aldrich (USA). A stock solution of CCCP was dissolved in DMSO 500 mM and stored at 4°C, and fresh ammonium sulphate (AS) was dissolved in water. Antibiotics were filtered a hydrophilic PVDF membrane with a 0.22 μm pore size.

### MIC determination

Bacteria were grown to log-phase. The MIC was performed by 2-fold dilutions of the antibiotics in 96-well polystyrene microtiter plates (Corning), the bacteria were incubated with indicated antibiotics at 37°C, overnight. The MIC was determined as the concentration of antibiotics that inhibit bacterial growth. All MICs were tested in duplicate at least three times. The MIC of each antibiotic for different bacteria is shown in Table 1.

**Table 1.** MIC (μg/ml) for strains used in this study.

	Antibiotics MIC (μg/ml)		
Strain	Kanamycin	Gentamicin	Amikacin
*A. baumannii* ATCC19606	8	4	4
*E. coli* ATCC 25922	8	2	2
*K. pneumoniae* ATCC700603	20	4	2
*M. smegmatis* MC^2^-155	4	2	2

### Persister cells isolation

Persisters can be induced in response to antibiotics, pre-treatment of *E. coli* with low levels of ciprofloxacin induced the formation of persisters to higher doses of ciprofloxacin [30]. Previous work has demonstrated that treatment the *E. coli* with 5 μg/ml ofloxacin for 3 h eliminates all susceptible non-persister cells [31]. For *A. baumannii* persister assays, bacteria were grown to stationary phase and diluted 50-fold with LB medium. Cultures were then treated with 5 μg/ml ciprofloxacin at 37°C for 4 h. Surviving cells were pelleted and suspended in M9 minimal medium. We verified that remaining cells were persisters by increasing the concentration of ciprofloxacin up to 125-fold MIC and noted no further decrease in viability (Figure S1).

### Antibiotic survival assay

To obtain exponential- and mid-stationary-phase cultures, overnight cultures (16 h) were diluted 1:1000 in fresh medium and grown into OD_600_ = 0.8 and 1.6, respectively. Cultures were centrifuged at 5000 rpm for 5 min, and the pellets were washed twice with PBS and re-suspended in M9 medium at OD_600_ = 0.1. For antibiotic treatment, bacteria were kept at the volumes of 1 ml in 12-well plate without shaking or 1 ml in sterilized glass tube with shaking. The cells were treated with kanamycin, gentamicin or amikacin with or without L-lysine in 37°C for 4 h. After treatment, 100 μl samples were serially diluted, and 10 μl aliquots were plated on LB agar plates. The results are averages from four biological replicates, and error bars represent standard deviations.

### Proton motive force quantification

Determination of PMF was performed using DiBAC_4_(3), as previously described [24,26]. Briefly, DiBAC_4_(3) was re-suspended in DMSO to form a 1 mM stock and then diluted to a final concentration of 1 mM. Bacteria were grown to stationary phase and were re-suspended in M9 minimal medium with OD_600_ = 0.3 for 6 h with various concentrations of L-lysine and/or Gen, Kan and Ami, as described above. DiBAC_4_(3) was added for an additional 10 min, and the cells were washed and analysed using BD FACSCANTO II Flow Cytometer for fluorescein isothiocyanate (FITC)-A fluorescence.

### ROS determination

ROS was measured by flow cytometry using 2′,7′-dichlorodihydrofluorescein diacetate (DCFH-DA). Exponential-phase cultures were washed twice with 1×PBS and re-suspended in M9 minimal medium, they were treated with Gen, Kan and Ami in the presence or absence of 20 mM L-lysine. Then, the cells were washed twice with PBS and incubated with 10 mM DCFH-DA at 37°C for 20 min. The cells were then washed twice with PBS, re-suspended in 200 μl PBS and analysed using a flow cytometer for FITC-A fluorescence.

### Ethidium bromide accumulation assay

The ethidium bromide (EB) accumulation assay was used to measure the florescence intensity with minor modifications [32]. Briefly, mid-log-phase cultures were washed with PBS containing 0.05% Tween 80 (PBST) and then re-suspended in M9 minimal media supplemented with or without L-lysine for 6 h. EB (1 μg/ml) was used for accumulation assays. In all assays, the cells were incubated in 96-well plates, and the analysis was performed at the indicated time points by excitation at 544 nm and emission at 590 nm on a FLUOstar OPTIMA Microplate Reader (BMG Labtech). All data were normalized to the time zero time point reading of each well.

### NAD^+^ and NADH measurements

*A. baumannii* culture was collected and diluted with M9 minimal media cultures to an OD_600_ = 0.3 and incubated with L-lysine at 37°C for 6 h. The NAD^+^/NADH assay was performed following the manufacturer’s protocol (EnzyChrom NAD/NADH Assay Kit, BioAssay Systems).

### Metabolon-based energy metabolism detection

Logarithmic phase cells were washed with PBS and re-suspended in M9 media with or without L-lysine. After 6 h incubation, the cells were pelleted (5 min at 140,00*g*, 4°C), washed with cold PBS, and snap frozen in liquid nitrogen. Sextuplicate samples were collected and sent for analysis by Metabolon-associated energy metabolism (Applied Protein Technology, Shanghai, China). A homogenate of 100 mg of sample mixed with 1 ml of cold methanol/acetonitrile/H_2_O (2:2:1, v/v/v) was sonicated at a low temperature (30 min/once, twice) and then centrifuged for 20 min (140,00*g*, 4°C). The supernatant was dried in a vacuum centrifuge. For LC-MS analysis, the dried samples were dissolved in 100 μl acetonitrile/water (1:1, v/v), adequately vortexed and then centrifuged (140,00 rpm, 4°C, 15 min). The supernatants were collected for the LC-MS/MS analysis. Analyses were performed using an UHPLC (1290 Infinity LC, Agilent Technologies) coupled to a QTRAP (AB Sciex 5500).

### Data Analysis and Statistics

FlowJo V10 was used for processing flow cytometric data, GraphPad prism was used to plot survival assays. All the data are analysed by GraphPad prism and shown as the mean ± SD of triplicate wells. Similar results were obtained in three independent experiments. *P < 0.05; **P < 0.01, ***P < 0.001. All figures were formatted with Adobe Illustrator.

## Results

### Exogenous L-lysine increases the susceptibility of A. baumannii to AGs

**Figure 1.**
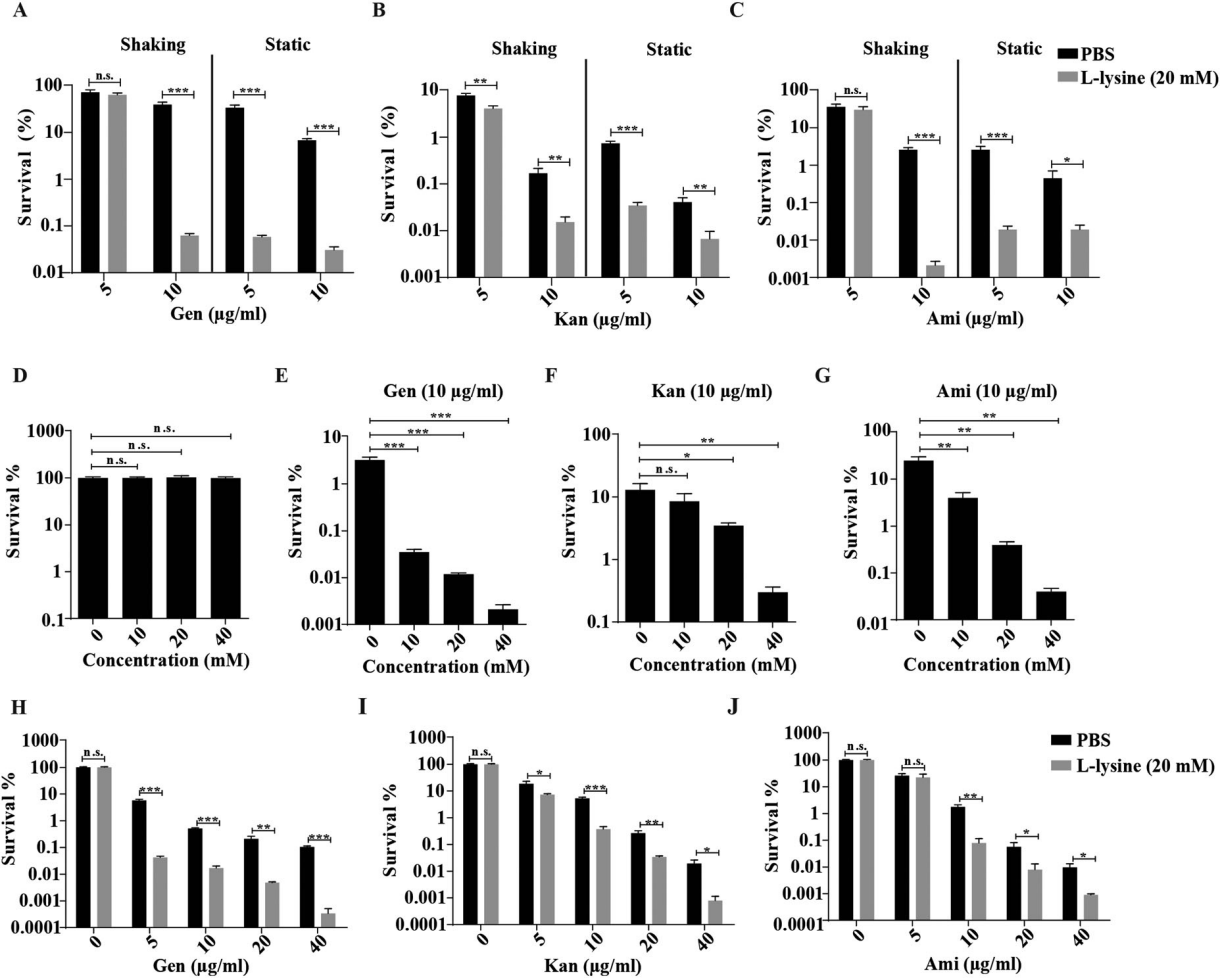
L-lysine increases the susceptibility of exponential-phase *A. baumannii* 19606 to aminoglycoside antibiotics, including gentamicin (Gen), kanamycin (Kan), and amikacin (Ami). (A–C) The survival of *A. baumannii* 19606 in the presence of or absence of 20 mM L-lysine and 5 μg/ml or 10 μg/ml Gen (A), Kan (B) and Ami (C) with shaking or static cultivation for 6 h. (D) Percentage survival of *A. baumannii* 19606 after treatment with different concentrations of L-lysine for 6 h. (E–G) *A. baumannii* 19606 culture was incubated with increasing concentrations of L-lysine in the presence of 10 µg/ml Gen (E), 10 µg/ml Kan (F) and 10 µg/ml Ami (G) for 6 h. (H–J). An exponentially growing *A. baumannii* culture was treated with increasing concentrations of Gen (H), Kan (I) or Ami (J) for 6 h in the presence of L-lysine (20 mM). The cultures were platted onto LB agar to determine the survival of bacteria.

It has been previously shown that the effect of L-arginine on AGs was more potent in anaerobic conditions, the effect of L-lysine on AGs against *A. baumannii* in different culture conditions was tested. We found that antibiotics cause less cell death on shaking-grown *A. baumannii* than bacteria cultured in static cultivation in the presence of L-lysine (Figure 1(A–C)). Although L-lysine promotes Kan against the bacteria, there is no potentiation of L-lysine on Gen and Ami at the finial concentration of 5 μg/ml when the bacteria cultured with shaking. However, this disappeared potentiation of L-lysine on antibiotics recovers when we raised the finial concentration of antibiotics into 10 μg/ml. In particular, the combination of L-lysine with AGs at the finial concentration of 5 μg/ml or 10 μg/ml induces comparable cell death of bacteria cultured in static cultivation. Thus, we treated the bacteria with AGs in the presence of L-lysine in static cultivation through all the experiments. Log-phase growing *A. baumannii* cultures were treated with AGs in the presence of or absence of L-lysine as indicated concentration that did not affect bacterial growth (Figure 1(D)). The survival of the bacteria is decreased in an L-lysine dose-dependent manner for AGs, including Gen (Figure 1(E)), Kan (Figure 1(F)) or Ami (Figure 1(G)). Next, we treated exponential-phase bacteria with antibiotics in the presence of 20 mM L-lysine, the survival of the bacteria is significantly reduced by treatment with Gen (Figure 1(H)), Kan (Figure 1(I)) and Ami (Figure 1(J)) in the presence of L-lysine. Moreover, the mortality of stationary-phase bacteria exposed to antibiotics in the presence of L-lysine is similar to the results observed using exponential-phase cultures (Figure S2). In these cases, antibiotic-mediated cell killing is more severe in the presence of L-lysine. These results prompt us to explore why the supplementary of L-lysine is useful for AGs against bacteria.

### L-lysine-induced PMF production is independent of TCA cycle

**Figure 2.**
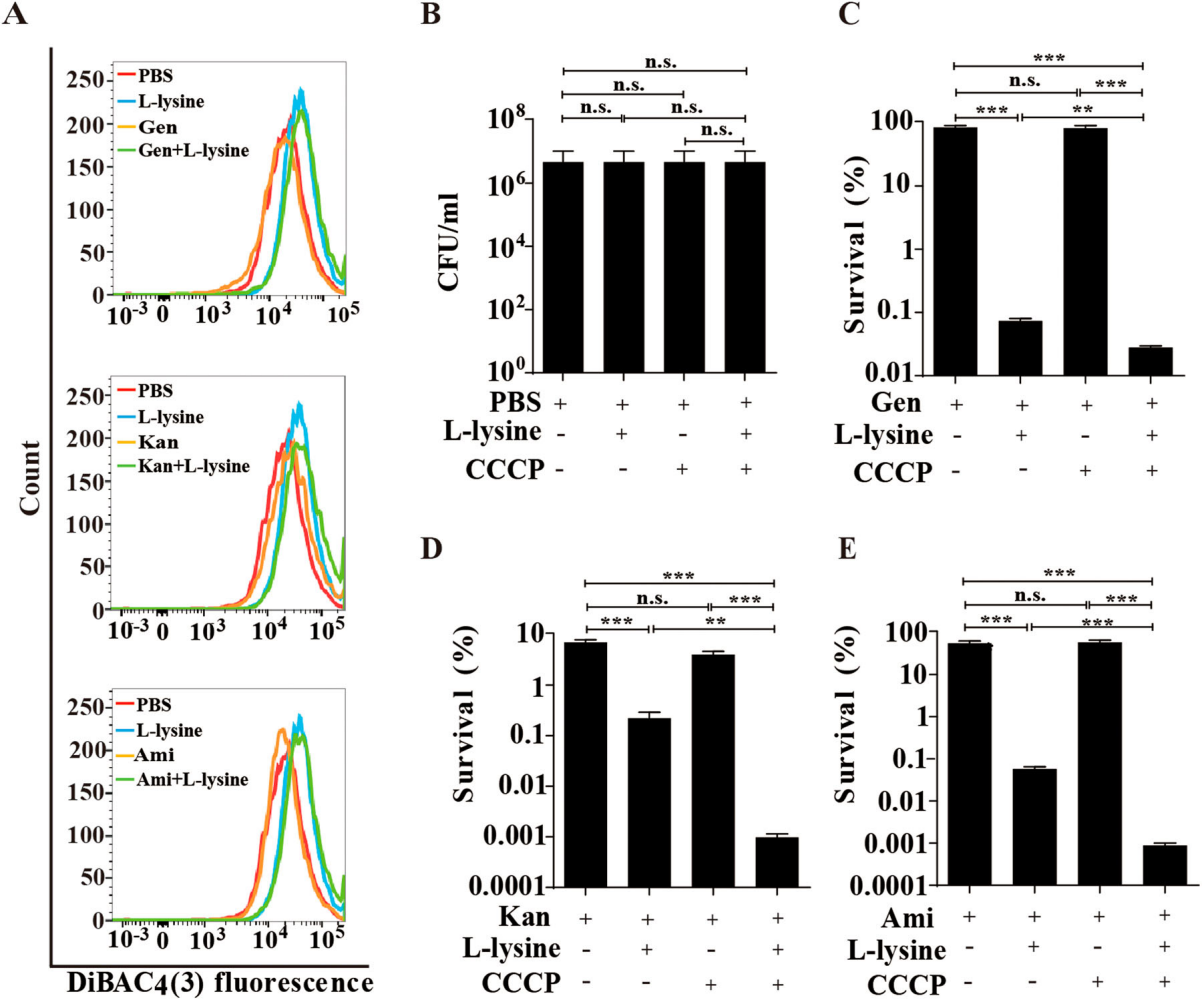
L-lysine increases PMF of *A. baumannii* which is independent on TCA cycle. (A) PMF in *A. baumannii* 19606 in the presence of 20 mM L-lysine and 10 μg/ml Gen, 10 μg/ml Kan and 10 μg/ml Ami, respectively. (B–E) Survival of exponential-phase *A. baumannii* 19606 in the presence or absence of 20 mM L-lysine and 20 µM CCCP (B), and in the presence of 10 μg/ml Gen (C), 10 μg/ml Kan (D), or 10 μg/ml Ami (E). Cultures were platted onto LB agar to determine the survival of bacteria.

Previous studies have reported that glucose or glucose plus alanine promotes the uptake of AGs by increasing the PMF [25,26]. We found that L-lysine promotes PMF production of *A. baumannii* in the presence or in the absence of antibiotics (Figure 2(A)). The electrical potential (ΔΨ) driven by tricarboxylic acid cycle (TCA) control is responsible for generation of PMF, which is known to drive the uptake of AGs [18]. To disrupt PMF on the L-lysine-enhanced antibiotics killing, we used CCCP, a proton ionophore that can abolish Δψ [33]. Consistent with previous studies, the addition of CCCP partially reduces antibacterial activity of AGs in the presence of L-lysine against *E. coli* (Figures S3A-D) and *M. smegmatis* (Figures S3E-H). Strikingly, *A. baumannii* displays high mortality by antibiotic killing when exposed to L-lysine in the presence of CCCP, suggesting that L-lysine-induced PMF is independent of ΔΨ (Figure 2(B–E)). To further determine whether L-lysine increased the PMF is associated with TCA control, the metabolomics was analysed using LC/MS/MS. Unsupervised hierarchical clustering and Z scores were used to rank metabolites whose abundance differed significantly in the bacteria in the presence of or absence of L-lysine (Figure S4A). However, the accumulation of metabolites associated with TCA is decreased in L-lysine-treated bacteria in comparison to bacteria alone (Figures S4B and D). In addition, the intracellular NADH and NAD^+^ levels were measured by the EnzyChrom NAD^+^/NADH Assay Kit after the bacteria were incubated with 20 mM L-lysine for 6 h. The concentration of NADH and NAD^+^ is lower in the L-lysine-treated bacteria than that in control group concentration. The NAD^+^/NADH ratio is increased in the L-lysine-treated bacteria due to a greater decrease in the NADH concentration (Figure S4C). These data suggest that L-lysine-induced PMF production is independent of TCA cycle.

### L-lysine induces transmembrane pH difference of PMF and promotes AGs uptake

**Figure 3.**
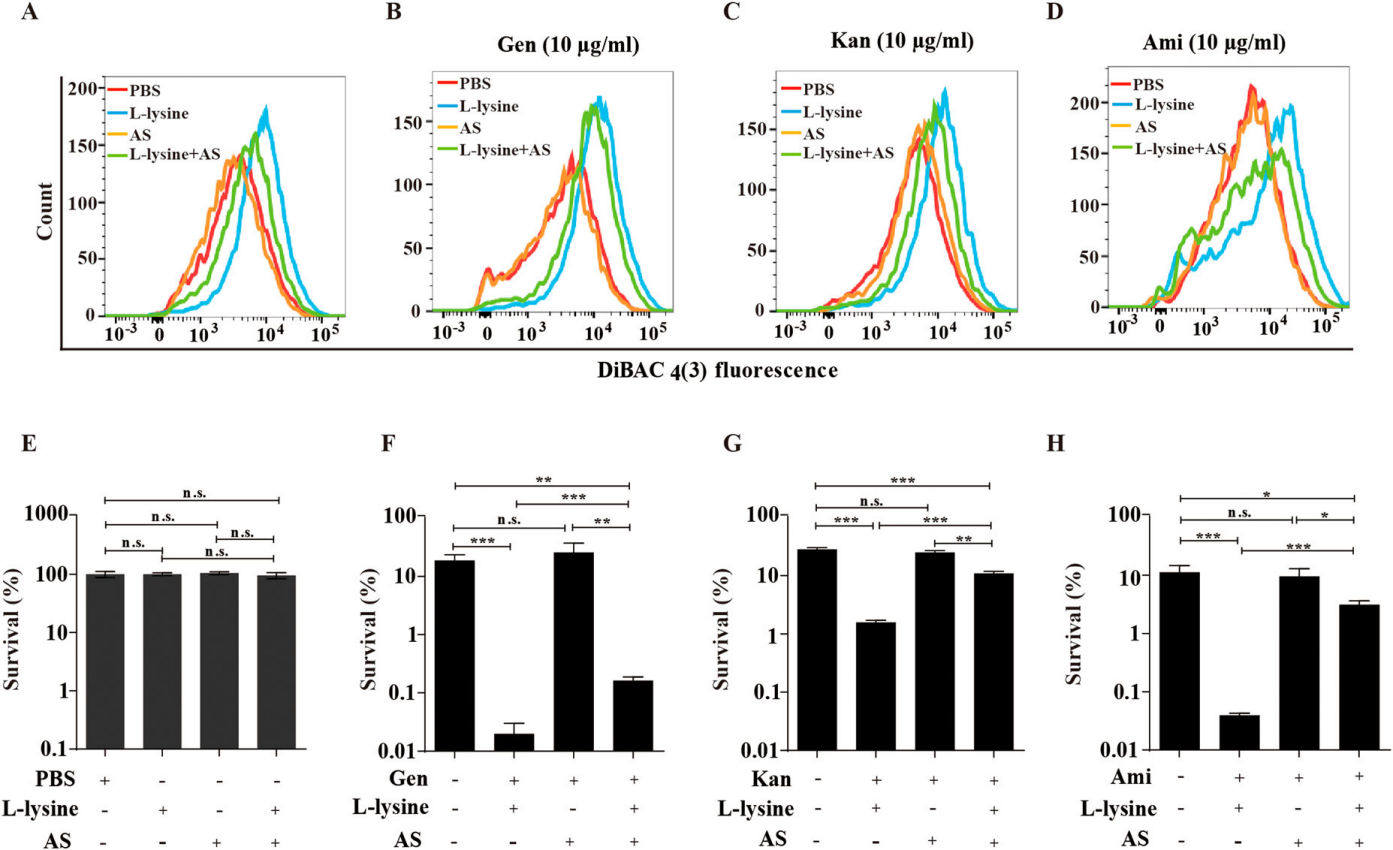
L-lysine-stimulated PMF production depends on transmembrane pH difference (ΔpH). (A–D) PMF in *A. baumannii* 19606 in the presence of or absence of 20 mM L-lysine and/or 10 mM AS (A), and in the presence of 10 µg/ml Gen (B), 10 µg/ml Kan (C), or 10 µg/ml Ami (D). (E–H) Percent survival of *A. baumannii* 19606 in the presence or absence of AS and when treated with L-lysine (E), and in the presence of 10 µg/ml Gen (F), 10 µg/ml Kan (G), or 10 µg/ml Ami (H). The cultures were platted onto LB agar to determine the survival of bacteria.

**Figure 4.**
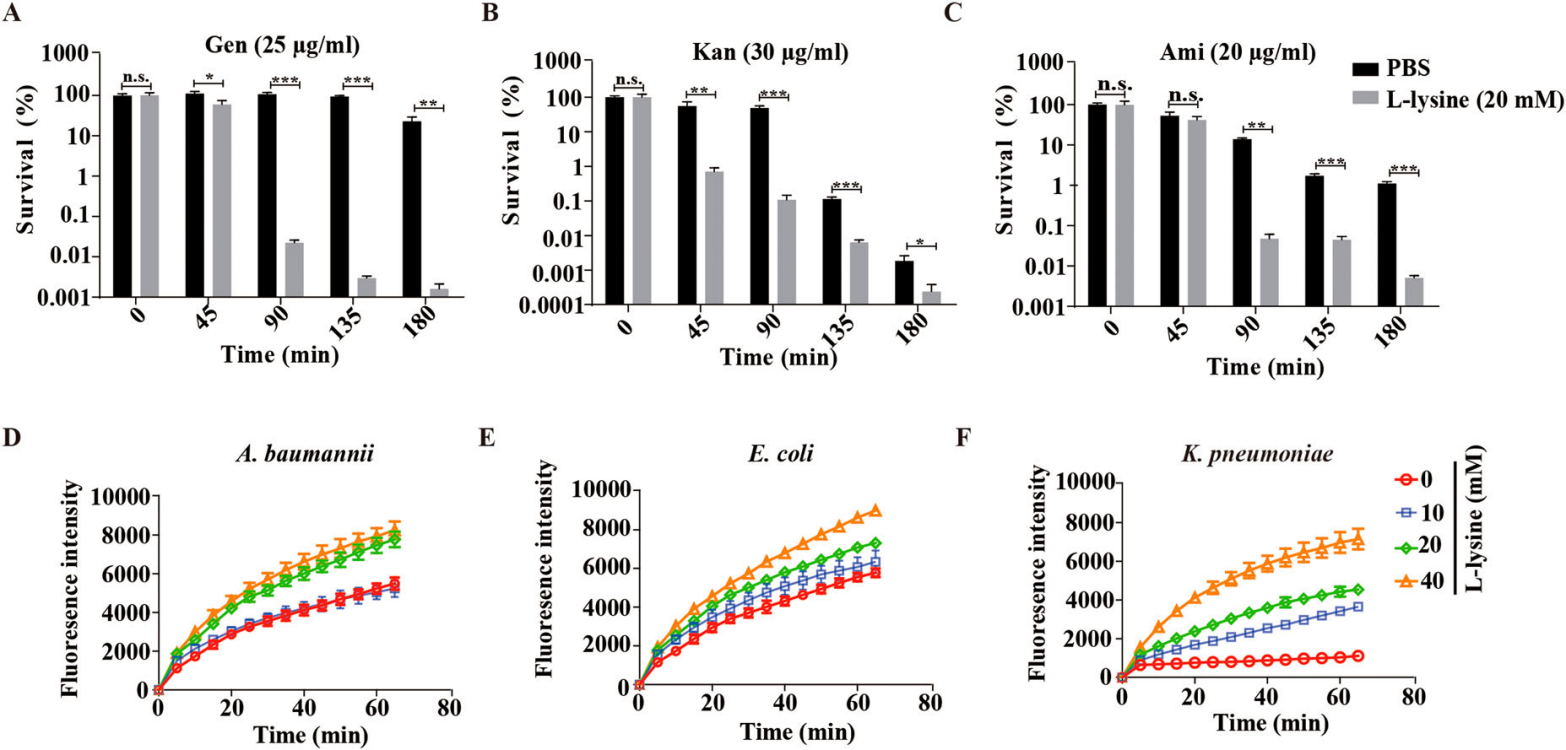
L-lysine treatment affects the cell permeability of bacteria. (A–C). Pretreatment of *A. baumannii* 19606 with 20 mM L-lysine for 3 h accelerated antibiotic killing of 25 μg/ml Gen (A), 30 μg/ml Kan (B), or 20 μg/ml Ami (C). (D–F) L-lysine increased the cell permeability of *A. baumannii* 19606 (D), *E. coli* ATCC 25922 (E) and *K. pneumoniae* ATCC700603 (F).

PMF includes both the Δψm and transmembrane pH difference (ΔpH) [33]. The uptake of tetracyclines, such as minocycline, is known to be driven by ΔpH of PMF [34]. We found that L-lysine promotes PMF production in the presence or in the absence of antibiotics, while it is reduced in the presence of ΔpH inhibitor AS (Figure 3A–D). Moreover, the addition of AS also partially recovers the survival of the bacteria from L-lysine and AGs treatment (Figure 3E–H). These results suggest that the importance of the ΔpH of PMF induced by L-lysine in increasing the uptake of AGs. The effectiveness of antibiotics mainly depends on their intracellular concentration. Mechanisms that lead to a higher drug permeability could be an alternative pathway to enhance susceptibility to antibiotics [35–38]. Then, we determined the effects of L-lysine on the cell permeability. Interestingly, the antibiotic killing enhanced by L-lysine can be detected after 45 min when *A. baumannii* was pretreated with L-lysine for 4 h (Figure 4(A–C)), while L-lysine has no effect on antibiotic killing within 3 h in the bacteria without L-lysine pretreating (Figure S5). L-lysine can increase the cell permeability of bacteria, including *A. baumannii* 19606 (Figure 4(D)), *E. coli* ATCC 25922 (Figure 4(E)) and *K. pneumoniae* ATCC700603 (Figure 4(F)). These results suggest that L-lysine increases cell permeability and antibiotic uptake.

### L-lysine promotes endogenous ROS to alter the susceptibility of A. baumannii to AGs

**Figure 5.**
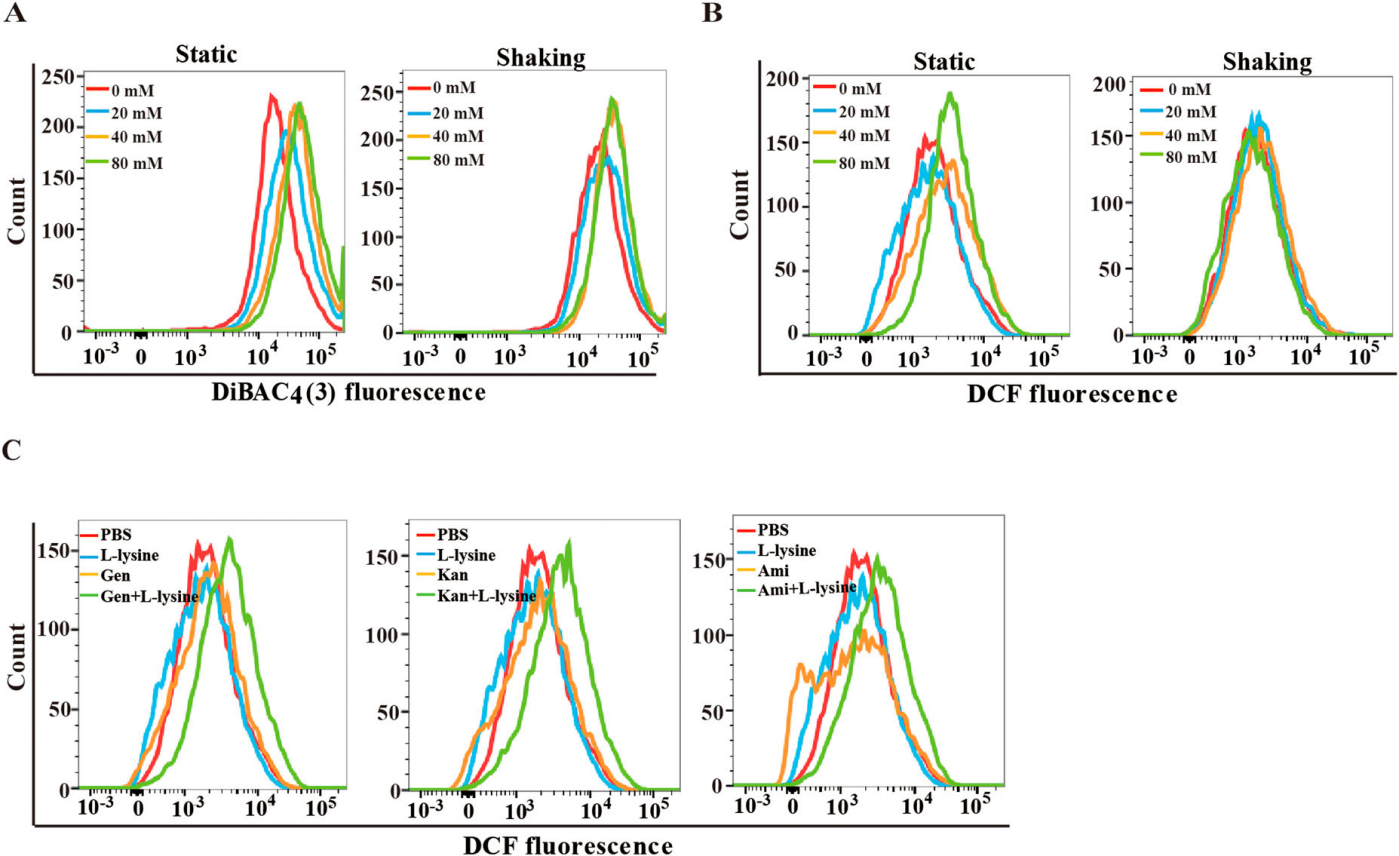
L-lysine regulates cooperatively PMF and intracellular ROS production. (A) PMF in *A. baumannii* 19606 in the presence of different concentration of L-lysine for 4 h with shaking or static cultivation. (B) ROS in *A. baumannii* 19606 in the presence of different concentration of L-lysine for 4 h with shaking or static cultivation. (C) ROS in *A. baumannii* 19606 in the presence of 20 mM L-lysine and 10 μg/ml Gen, 10 μg/ml Kan and 10 μg/ml Ami, respectively. The cultures were platted onto LB agar to determine the survival of bacteria.

To examine this difference in bacterial killing, we compared the PMF in L-lysine-treated bacteria with shaking or static cultivation. L-lysine induces PMF in both shaking and static cultured bacteria, although L-lysine causes slightly higher PMF in static cultured bacteria than shaking cultured bacteria (Figure 5(A)). These data suggest there could be other underlying mechanism in which L-lysine enabled antibiotic killing. As antibiotics kill bacteria, in part, by inducing ROS [39–41], we reasoned that L-lysine might also target the microbial ROS production to potentiate antibiotic activity. We next compared the production of ROS in *A. baumannii* cultured in static cultivation and shaking in the presence of different concentration of L-lysine. ROS production is only found in static cultured *A. baumannii* treated by L-lysine at the concentration of 80 mM (Figure 5(B)). Interestingly, higher ROS accumulation in *A. baumannii* was induced by 20 mM L-Lysine plus antibiotic treatment in static cultivation (Figure 5(C)). These results suggested that L-lysine not only accelerated PMF production but also altered ROS accumulation under antibiotic stress.

### L-lysine potentiates AGs against clinically relevant multidrug-resistant bacteria and other bacteria

**Figure 6.**
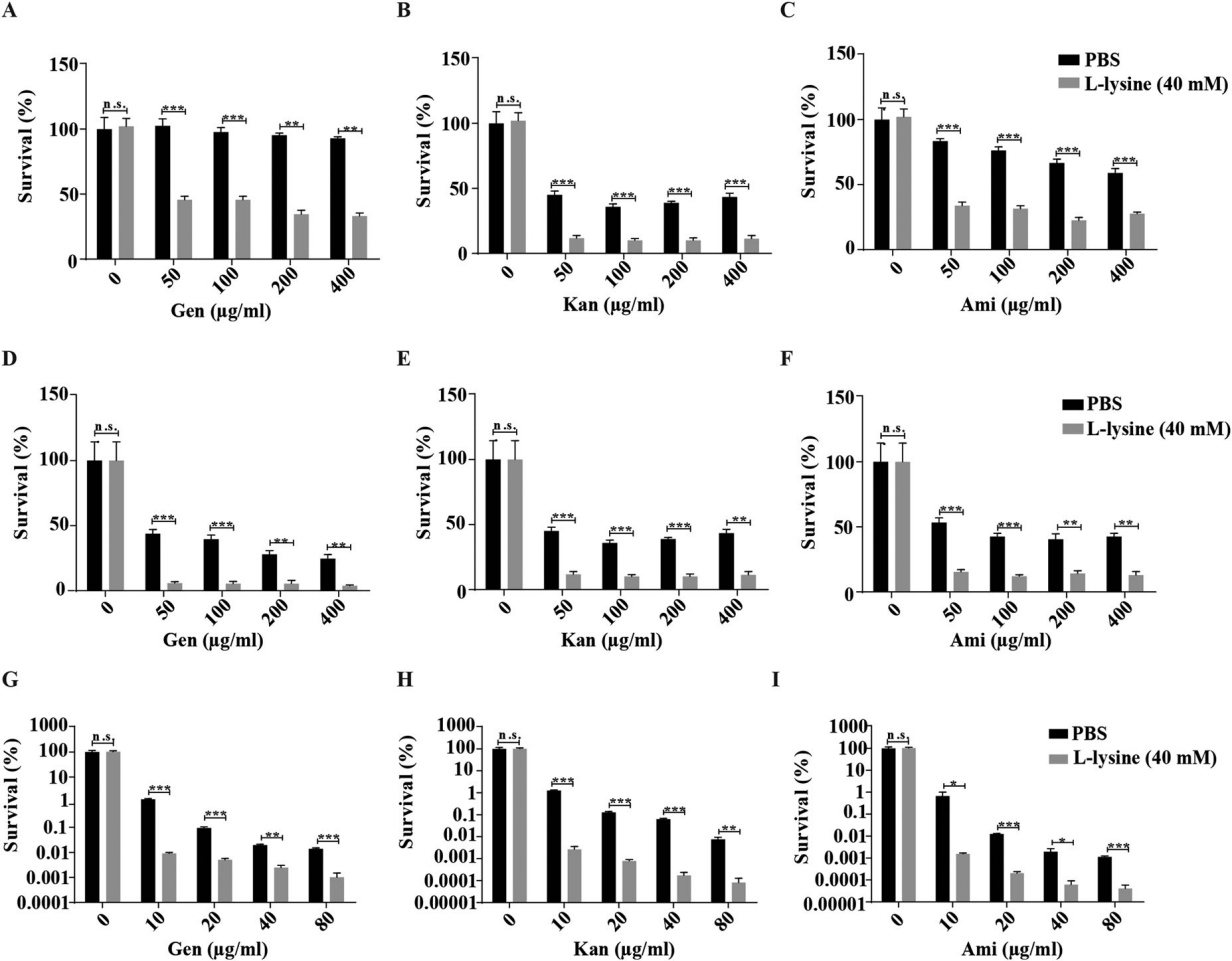
L-lysine elevates the susceptibility of clinically isolated multidrug resistance *A. baumannii* and persisters to aminoglycoside antibiotics. (A–C) Clinically isolated multidrug resistance *A. baumannii* CRAb16010214 was treated with 40 mM L-lysine with an increasing concentration of Gen (A), Kan (B) and Ami (C), respectively. (D–F) The survival of another clinically isolated multidrug resistance *A. baumannii* CRAb18030945 in the presence of 40 mM L-lysine and Gen (D), Kan (E) and Ami (F), respectively. (G–I) Persisters were treated with different doses of Gen (G), Kan (H) and Ami (I) in the presence of 40 mM L-lysine. Clinically isolates and persisters were mixed L-lysine and antibiotics as indicated overnight. The cultures were platted onto LB agar to determine the survival of bacteria.

**Figure 7.**
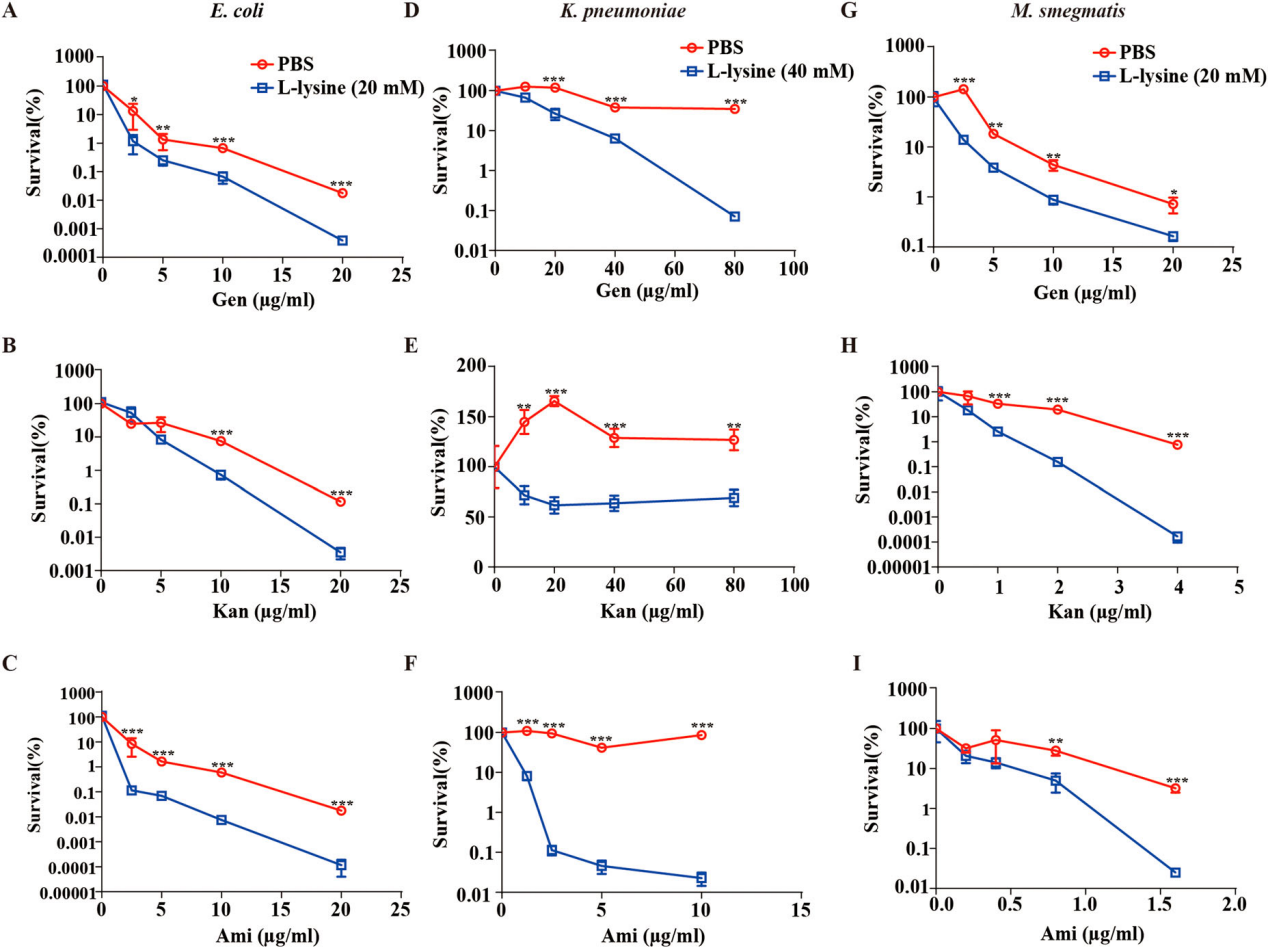
L-lysine potentiates aminoglycosides against other Gram-negative and Gram-positive bacteria. (A–C) *E. coli* ATCC 25922 was treated with 20 mM L-lysine with an increasing concentration of Gen (A), Kan (B) and Ami (C). (D–F) The survival of Gram-negative baterium *K. pneumoniae* ATCC700603 in the presence of 40 mM L-lysine and Gen (D), Kan (E) and Ami (F). (G–I) Percent survival of Gram-positive bacterium *M. smegmatis* MC^2^-155 in the presence of 20 mM L-lysine and Gen (G), Kan (H) and Ami (I). Logarithmic growth phase bacteria cells were re-suspended in M9 medium with an OD_600_ of 0.2. The re-suspended cells were mixed with L-lysine plus antibiotics as indicated at 37°C for 4 h. For the *E. coli* ATCC 25922 and *K. pneumoniae* ATCC700603, cultures were platted onto LB agar to determine the survival of bacteria. For *M. smegmatis* MC^2^-155, cells were plotted onto 7H9 agar to determine the survival of bacteria.

Multidrug-resistant bacteria and persister cell-related infections are a great concern in clinical facilities [42–44], and it would be clinically useful if multidrug-resistant bacteria and persisters were more susceptible to killing by AGs in the presence of L-lysine. *A. baumannii* CRAb18030945 and CRAb16010214 isolated from patients are two multidrug-resistant strain that confers resistance to amikacin, ampicillin\sulbactam cefepime, ceftazidime, ceftriaxone, ciprofloxacin, gentamicin, imipenem, levofloxacin and piperacillin/tazobactam (Table S1). We identified L-lysine act in a synergistic manner with AGs against *A. baumannii* (Figure 1(H–J)), so two MDR *A. baumannii* strains were co-incubated with L-lysine and challenged with different AGs, because L-lysine increased the efficiency of AGs against CRAb18030945 (Figure 6(A–C)) and CRAb16010214 (Figure 6(D–F)). Persisters are pre-existing and formed randomly in microbial populations that are extremely tolerant to antibiotics [45,46]. Joseph Bigger originally referred a small subpopulation of *Staphylococcus aureus* survived from a lethal dose of penicillin as persisters [47]. Bacterial persisters have been shown to be highly tolerant to antimicrobials and have been reported to be the cause of persistent and difficult-to-treat infections [42]. When combined with AGs and L-lysine, the frequency of *A. baumannii* persisters was decreased 25-fold in the presence of antibiotic plus L-lysine (Figure 6(G–I)), suggesting that L-lysine promotes the antibiotic susceptibility of both multidrug-resistant clinical isolates and bacterial persisters. In addition to *A. baumannii*, other relevant Gram-negative bacteria, including *E. coli* (Figure 7(A–C)) and *K. pneumoniae* (Figure 7(D–F)), and a Gram-positive bacterium *M. smegmatis* (Figure 7(G–I)), are susceptible to killing by AGs with L-lysine. Together, L-lysine by itself is incapable of preventing pathogenic infections, while it will enhance the action of AGs to combat pathogenic infections.

## Discussion

The ever-increasing incidence of antibiotic-resistant infections combined with a weak pipeline of new antibiotics has created a global public health crisis [48]. Accordingly, novel strategies for enhancing antibiotic arsenal are needed. AGs have shown their efficacy in the treatment of Gram-positive and -negative infections, while the emergence of AGs-resistant bacteria requires us to develop additional ways to combat antibiotic resistance [13]. A combination of compounds that enhance drug susceptibility and antibiotics represent a promising, effective and feasible way to improve the efficacy of existing antibiotics [49–51]. Here, we identified that exogenous L-lysine serves as a non-toxic adjuvant of AGs to kill *A. baumannii* (Figure 1), especially multidrug-resistant *A. baumannii* (Figure 6(A–F)) and persisters (Figure 6(G–I)). Similar results were observed in other Gram-negative bacteria (*K. pneumoniae* and *E. coli*) and a Gram-positive bacterium (*M. smegmatis*) (Figure 7).

The antimicrobial activity of AGs is enhanced by PMF via Δψ or ΔpH [18,52]. Fructose or mannitol [25] and alanine and/or glucose [26] stimulates PMF production relies on an increase in Δψ. Consistent with previous study [26], CCCP reduces L-lysine-mediated AGs against *E. coli* (Figures S3A-D) and *M. smegmatis* (Figures S3E-H). Unfortunately, the mortality of *A. baumannii* exposed to AGs in the presence of CCCP and L-lysine is higher than the addition of AGs and L-lysine, which is not due to the toxicity of CCCP itself (Figure 2(B-F)). Studies have shown that an excess of multidrug efflux pumps in *A. baumannii* is responsible for acquired multidrug [53,54]. CCCP has been reported to serve as efflux pumps inhibitor to reduce the susceptibility of *A. baumannii* to antimicrobial agents [55]. Therefore, we supposed efflux pumps inhibitor CCCP cooperates L-lysine to promote AGs against *A. baumannii*. Our results also demonstrated that L-lysine mediates PMF production via transmembrane ΔpH rather than Δψ (Figure 3). AS, an inhibitor of transmembrane pH, is observed to decrease L-lysine-induced PMF production significantly. The mortality of *A. baumannii* towards AGs plus L-lysine is decreased in the presence of AS (Figure 3). Together, our results implied that L-lysine increases ΔpH of PMF, resulting in antibiotics uptake and accumulation of antibiotics (Figure 4). Although a similar result demonstrated that the pH-mediated L-arginine effect probably relies on transmembrane ΔpH difference rather than on Δψ, the mechanistic aspects of this effect are still currently under investigation.

Moreover, L-lysine-mediated mortality of *A. baumannii* in the presence of AGs is more potent in static cultivation than shaking, while PMF production is comparable in both conditions (Figure 5(A)). We speculated that other contributing factors may present in L-lysine-induced AGs potentiation against *A. baumannii* in static cultivation. ROS accumulation within bacteria can trigger cell death or arrest cell growth via damaging proteins, DNA, RNA, and membrane lipids [56,57]. An increase in endogenous microbial ROS can increase the efficiency of antibiotic killing and is a well-known strategy against both Gram-positive and -negative bacteria [58]. Most of the bactericidal antibiotics that target *E. coli* induce lethality by a common mechanism despite having different primary targets [40,59–62]. Intracellular ROS production is induced in *A. baumannii* in the presence of higher concentration of L-lysine (Figure 5(B)), while ROS is quickly accumulated in *A. baumannii* treated with AGs in the presence of lower concentration of L-lysine in static cultivation (Figure 5(C)). Nevertheless, it is possible assumed that L-lysine-induced PMF production promotes intracellular AG accumulation, contributing to cellular ROS production.

L-lysine, one of the essential amino acids, which is necessary for many vital processes and needs to be obtained from dietary sources. It should be mentioned that most people can take L-lysine up to 3 g (about 20 mM) per day without any side effects (https://www.medicalnewstoday.com/articles/324019.php). We provided evidence that L-lysine (20 mM) is enough to enhance the in vitro antimicrobial activity AGs. However, the efficacy and the safety for the dose L-lysine supplement demands further investigation *in vivo*. In summary, this work established a strategy for eradicating bacterial infections and highlighted L-lysine serves as a promising adjuvant candidate of AGs or antibiotics against bacterial infection, especially clinical multidrug resistant bacteria or persister.

## Supplementary Material

Supplemental Material
