# Supplementary material for: L-lysine potentiates aminoglycosides against Acinetobacter baumannii via regulation of proton motive force and antibiotics uptake
Source: Emerg Microbes Infect. 2020 Mar 20;9(1):639–50. doi: 10.1080/22221751.2020.1740611 (PMC7144275; doi:10.1080/22221751.2020.1740611)
Supplement: Supplemental Material [file TEMI_A_1740611_SM8759.zip › Table S1.docx]

Table S1. Antibiotics resistance characters of *A. baumannii* CRAb18030945 and CRAb16010214

|  | MIC(μg/ml) | |
| --- | --- | --- |
| antibiotics | CRAb18030945 | CRAb16010214 |
| amikacin | ≥64 | ≥64 |
| ampicillin\sulbactam | ≥32 | ≥32 |
| cefepime | ≥64 | ≥64 |
| ceftazidime | ≥64 | ≥64 |
| ceftriaxone | ≥64 | ≥64 |
| ciprofloxacin | ≥4 | ≥4 |
| gentamicin | ≥16 | ≥16 |
| imipenem | ≥16 | ≥16 |
| levofloxacin | ≥8 | ≥8 |
| piperacillin/tazobactam | ≥128 | ≥128 |

Drug sensitive data obtained from clinical laboratory of Chongqing General Hospital.
