# Supplementary figures and images for: L-lysine potentiates aminoglycosides against Acinetobacter baumannii via regulation of proton motive force and antibiotics uptake
Source: Emerg Microbes Infect. 2020 Mar 20;9(1):639–50. doi: 10.1080/22221751.2020.1740611 (PMC7144275; doi:10.1080/22221751.2020.1740611)

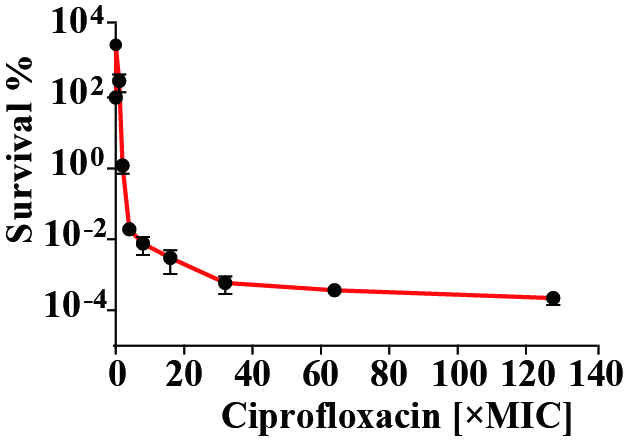

Supplement: Supplemental Material [file TEMI_A_1740611_SM8759.zip › Figure S1.tif]

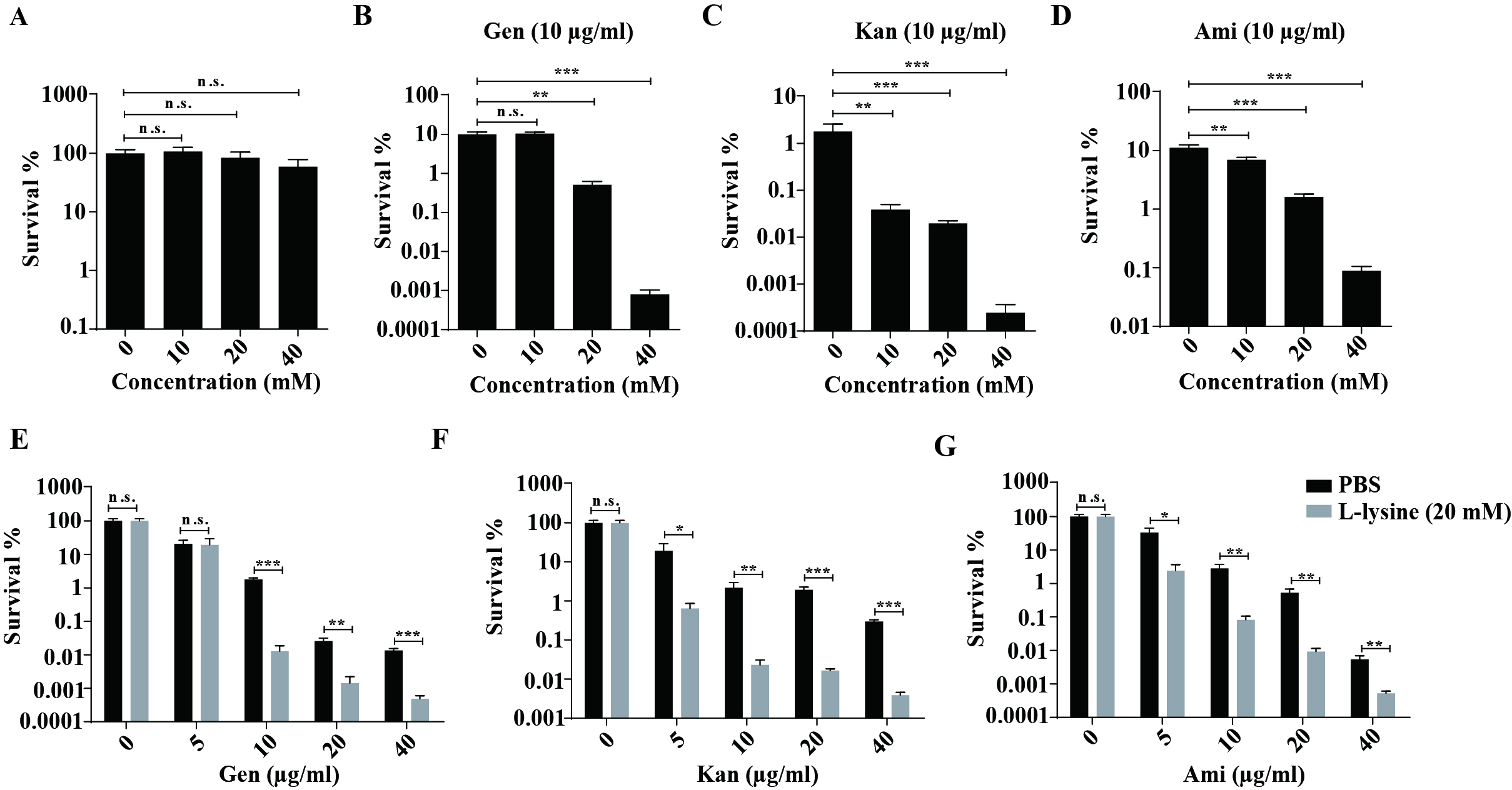

Supplement: Supplemental Material [file TEMI_A_1740611_SM8759.zip › Figure S2.tif]

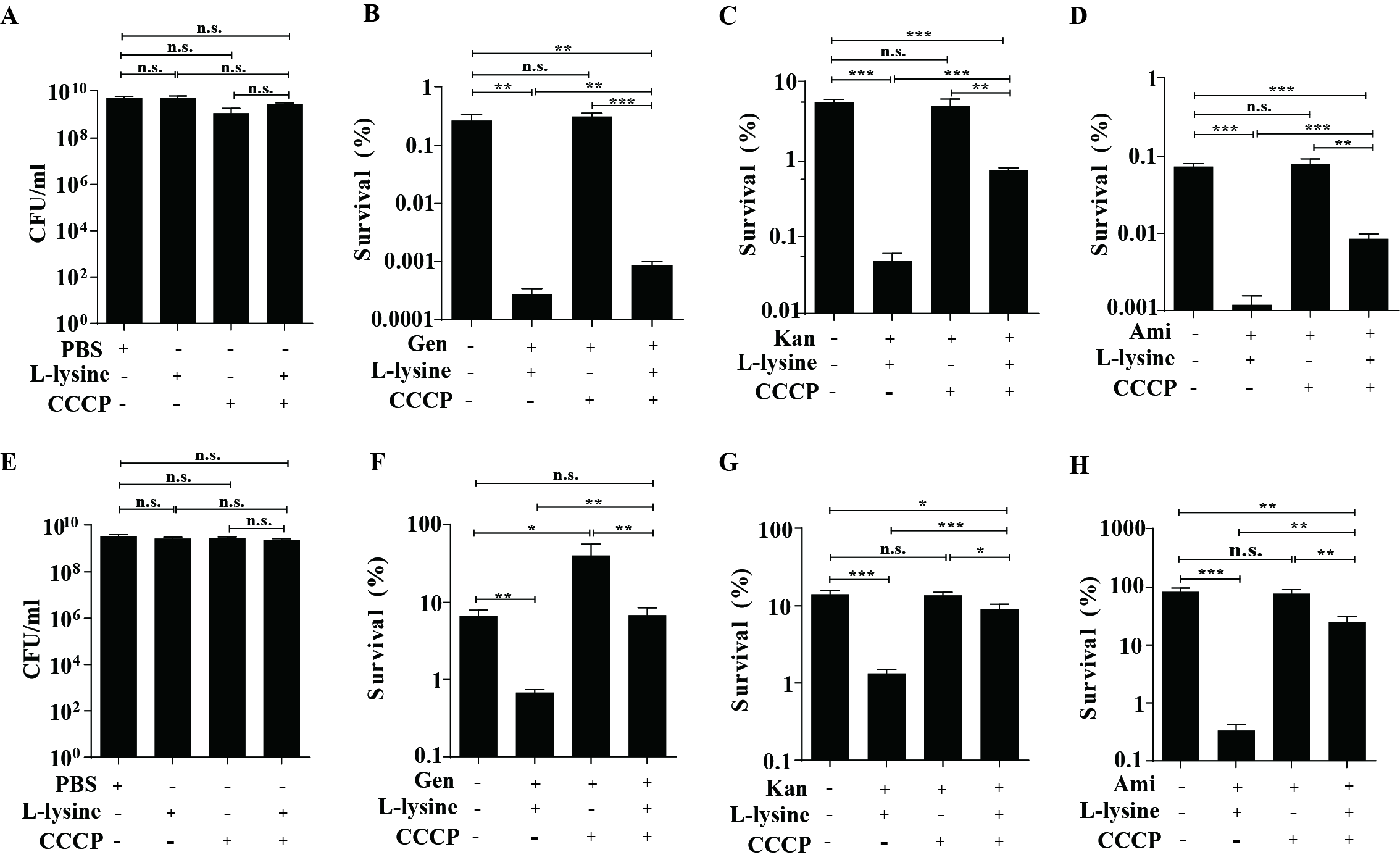

Supplement: Supplemental Material [file TEMI_A_1740611_SM8759.zip › Figure S3.tif]

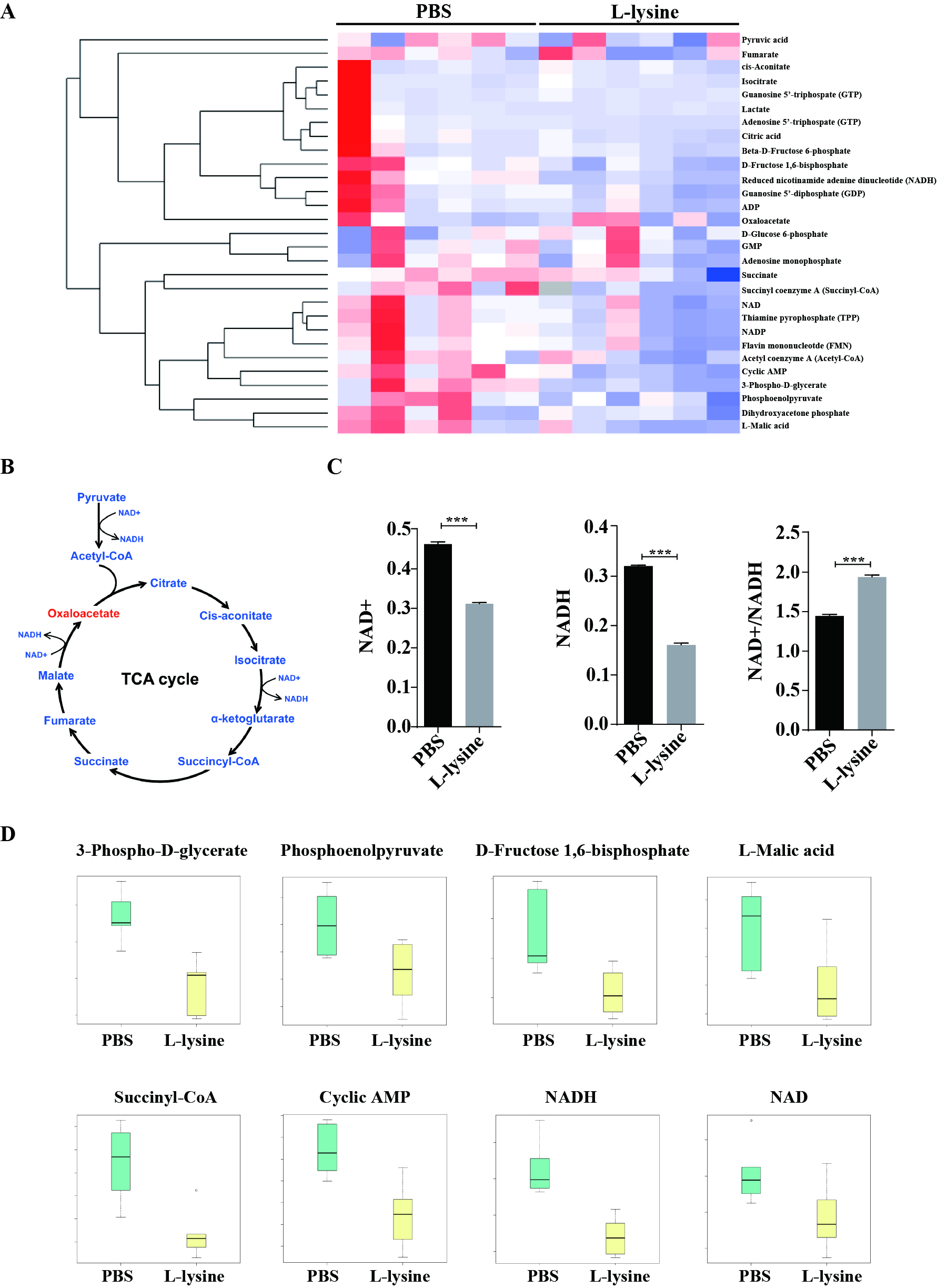

Supplement: Supplemental Material [file TEMI_A_1740611_SM8759.zip › Figure S4.tif]

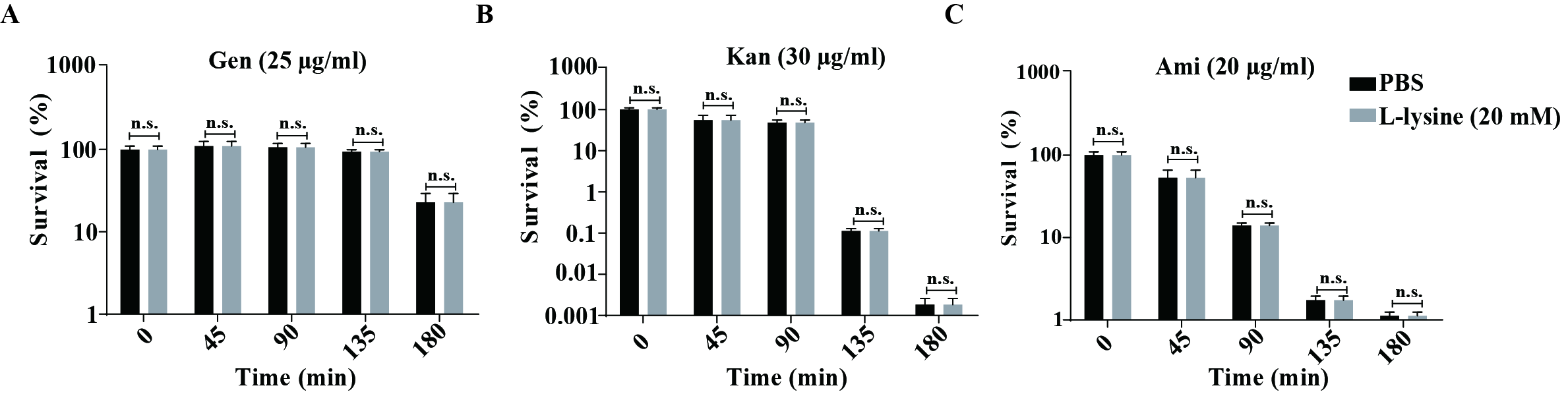

Supplement: Supplemental Material [file TEMI_A_1740611_SM8759.zip › Figure S5.tif]
